# Behavioral Factors Related to Participation in Remote Blood Pressure Monitoring Among Adults With Hypertension: Cross-Sectional Study

**DOI:** 10.2196/56954

**Published:** 2024-12-23

**Authors:** Chinwe E Eze, Michael P Dorsch, Antoinette B Coe, Corey A Lester, Lorraine R Buis, Karen B Farris

**Affiliations:** 1 Department of Clinical Pharmacy, College of Pharmacy, University of Michigan, Ann Arbor, MI, United States; 2 Department of Family Medicine, University of Michigan, Ann Arbor, MI, United States

**Keywords:** remote blood pressure monitoring, telemonitoring, hypertension, blood pressure, technology, health behaviors, quantitative, cross-sectional study, United States, lack of awareness, health information, health provider, electronic communication channels, adult, aging, mobile phone

## Abstract

**Background:**

Remote blood pressure (BP) monitoring (RBPM) or BP telemonitoring is beneficial in hypertension management. People with hypertension involved in telemonitoring of BP often have better BP control than those in usual care. However, most reports on RBPM are from intervention studies.

**Objective:**

This study aimed to assess participant characteristics and technology health behaviors associated with RBPM participation in a wider population with hypertension. This study will help us understand the predictors of RBPM participation and consider how to increase it.

**Methods:**

This was a quantitative, cross-sectional survey study of people with hypertension in the United States. The inclusion criteria included people aged ≥18 years with a hypertension diagnosis or who self-reported they have hypertension, had a prescription of at least one hypertension medication, understood the English language, and were willing to participate. The survey included demographics, technology health behaviors, and RBPM participation questions. The survey was self-administered on the Qualtrics platform and followed the CHERRIES (Checklist for Reporting Results of Internet E-Surveys) checklist. The primary dependent variable was participation in RBPM.

**Results:**

In total, 507 people with hypertension participated in the survey. The mean age for all respondents was 60 (SD 14.7) years. The respondents were mostly female (306/507, 60.4%), non-Hispanic (483/507, 95.3%), and White (429/507, 84.6%). A little over half of the respondents reported having had hypertension for 5 years or more (287/507, 56.6%). About one-third of participants were aware of RBPM (165/507, 32.5%), and 11.8% (60/507) were enrolled in RBPM. The mean age of those engaging in RBPM and non-RBPM was 46.2 (SD 14.7) and 62 (SD 13.7) years, respectively. The most common reasons for not participating in RBPM were because their health provider did not ask the participant to participate (247/447, 55.3%) and their lack of awareness of RBPM (190/447, 42.5%). Most respondents in the RBPM group measure their BP at home (55/60, 91.7%), and 61.7% (37/60) engage in daily BP measurement, compared with 62.6% (280/447) and 25.1% (112/447), respectively, among the non-RBPM group. A greater number of those in the RBPM group reported tracking their BP measurements with mobile health (mHealth; 37/60, 61.7%) than those in the non-RBPM group (70/447, 15.6%). The electronic health records or patient portal was the most common channel of RBPM communication between the respondents and their health care providers. The significant predictors of participation in RBPM were RBPM awareness (adjusted odds ratio [AOR] 34.65, 95% CI 11.35‐150.31; *P*<.001) and sharing health information electronically with a health provider (AOR 4.90, 95% CI 1.39‐21.64; *P*=.01) among all participants. However, the significant predictor of participation in RBPM among participants who were aware of RBPM was sharing health information electronically with a health provider (AOR 6.99, 95% CI 1.62‐47.44; *P*=.007).

**Conclusions:**

Participation in RBPM is likely to increase with increased awareness, health providers’ recommendations, and tailoring RBPM services to patients’ preferred electronic communication channels.

## Introduction

### Background

Remote blood pressure (BP) monitoring (RBPM) or BP telemonitoring is an important hypertension management strategy that involves electronic transfer of self-measured BP from the patient’s home to their doctor or doctor’s office with subsequent feedback based on the transmitted BP measurements [1]. RBPM offers many benefits to both patients and health care providers [1-3]. The patients can save time and cost of health care by reducing clinic visits, being more engaged in their disease management, and gaining a better understanding of their hypertension and how to keep it under control. Health care providers can follow their patients more closely, make timely health decisions, and provide improved quality of care to their patients. Various studies have demonstrated greater improvement in BP control among patients engaged in RBPM compared with those in usual care [1,2,4-8]. However, to reap the benefits of RBPM, patients need to engage in the RBPM program [9-11]. Patients’ engagement in RBPM depends on various factors which may be technology-, health system-, or patient-related. The technology needs to be simple and user-friendly. The health system is responsible for making these technological services available with adequate resources and manpower [12,13].

Patient-related factors that may influence RBPM engagement include patient demographics, technology health behaviors, poor electronic health literacy, lack of understanding of the risks associated with uncontrolled hypertension, lack of access to simple adequate technologies, privacy concerns, health data integrity, and security [2,5,12,14-16]. Technology health behaviors refer to technology-related actions taken by patients to improve their health. Technology health behaviors, such as electronic health information sharing between patients and their health care providers, have helped improve communication and decision-making between both parties [17]. The health information sharing could be done through the electronic health portals or mobile health (mHealth) devices, including tablet computers and mobile phones. Adults with one or more chronic disease conditions including hypertension, are more likely to use mHealth to access Web-based health support from their health care providers compared with those without any chronic diseases [18]. Patients with chronic diseases are also more likely to track health goals electronically, make health decisions, and hold discussions with their health care providers based on electronically found health information [19]. A study on people with hypertension who responded to the Health Information National Trends Survey (HINTS) found that patients who are already engaging with their health care providers electronically via email or the internet have higher odds of also communicating through SMS text messages with them compared with those who are not [20]. These studies, however, are not specific to RBPM.

Most studies on RBPM are intervention studies. There is a lack of data on the prevalence of RBPM participation among people with hypertension in everyday life situations. Patient-related factors that influence engagement in RBPM have been driven mostly by qualitative studies [21-26] and less by the quantitative assessment of patients’ characteristics to identify predictors of engagement [27]. Quantitative assessment of technology health behaviors focused on RBPM participation are therefore warranted.

### Objectives

The objectives of this study were to (1) assess participation and nonparticipation in RBPM among adults with hypertension and (2) assess patients’ characteristics and technology health behaviors that predict participation in RBPM. We hypothesized that participation in RBPM is associated with patients’ characteristics and technology health behaviors.

## Methods

### Design

The study was a quantitative cross-sectional survey of patients with hypertension in the United States.

### Ethical Considerations

The study was approved by the University of Michigan Institutional Review Board with the approval number HUM00205760. Informed consent and the ability to opt out of the study were provided to the participants before they could participate in the study. Anonymized data containing no identifiable personal information were collected from the participants. The participants were compensated with the amount they agreed on with Qualtrics before entering the study.

### Participants

The participants were recruited and surveyed using a web-based Qualtrics panel. Qualtrics panel members are real people whose names, addresses, and date of birth have been validated. Qualtrics recruits them from all over the United States to participate in surveys. The inclusion criteria were patients aged ≥18 years who self-reported a hypertension diagnosis, had at least one prescription hypertension medication, understood the English language, and were willing to participate. Exclusion criteria included active cancer, diagnosis of cognitive impairment, or having been to the intensive care unit in the past 6 months. We used the exclusion criteria because people with active cancer, cognitive impairment, or who were recently in intensive care unit were more likely to be closely monitored by their health care providers and may not provide the general RBPM practice obtainable in the hypertension population.

### Sample Size

With the 47.3% adult population with hypertension in the United States in 2021 [28], using 5% type 1 error (*P*=.05), the minimum sample size required to estimate participation in RBPM was 383 participants [29]. A minimum sample size of 500 has been recommended for the detection of differences between sample estimates and the population in observational studies involving logistic regression [30]. We therefore recruited 507 participants with hypertension using the quota sampling explained in the recruitment section.

### Recruitment

The participants were recruited using the Qualtrics panel. Based on our preliminary study of a secondary analysis of the 2018 HINTS 5 Cycle 2, age and education were associated with electronic health information seeking among respondents with hypertension. Therefore, we used a priori quota sampling based on age and educational levels. To ensure adequate representation within the age and education groups, the following proportions from the analysis of HINTS 5 Cycle 2 respondents with hypertension were used: less than 50 years (15%), 50‐74 years (64%), 75 years and above (21%), less than college education (32%), some college education (34%), college graduate, and above (34%). The study, extent of participation, and incentive for participation, were described to the participants meeting the inclusion criteria. The participants were screened for study inclusion eligibility, and consented before they completed the survey. The participants were compensated based on the amount they agreed upon before entering the survey.

### Data Collection

The web-based survey had 3 parts including demographics and clinical characteristics questions, BP self-monitoring and telemonitoring behavior questions, and technology health behavior questions. The survey was self-administered and took about 15 minutes to complete. The survey was in the field from November to December 2021. More information on the survey using the CHERRIES (Checklist for Reporting Results of Internet E-Surveys) checklist [31] is presented in Checklist 1.

### Demographics and Clinical Characteristics

We collected respondents’ demographics and clinical characteristics including age, sex, ethnicity, race, educational level, marital status, income, clinic distance from residence, residential area, general health status, comorbidities, length of time since being diagnosed with hypertension, number of hypertension medications, number of other medications different from the hypertension medications, BP control status, and the last measured systolic and diastolic BP values.

### BP Self-Monitoring and Telemonitoring Behaviors

We collected data on respondents’ routine BP monitoring and tracking behaviors, RBPM awareness, participation and telemonitoring strategies used. We provided a description of RBPM to respondents like this: “Remote blood pressure monitoring is a newer way to keep control of your blood pressure from home. In remote blood pressure monitoring, you measure your BP at home and send the readings or measurements to your health care provider through an electronic means. You may or may not receive feedback from your provider electronically. You still visit the office for problems or yearly check-ups.” The questions used in the data collection were formulated from activities that patients usually must do in home BP monitoring and RBPM programs [4-7]. We also asked those not participating in RBPM to state their reasons for not participating and their likelihood of participation if RBPM were offered to them (very likely, somewhat likely, neither likely nor unlikely, somewhat unlikely, very unlikely).

### Technology Health Behaviors

For this survey section, we adapted variables from HINTS [32] focusing on technology ownership and use within a 12-month recall period. We also asked for patient-provider communication or interaction preferences regarding BP management, whether through in-person clinic visits or electronic means including email, phone calls, SMS text messages, electronic health records, and video visits. Participants who responded “no” to ownership of a home BP monitoring device were asked to state their reasons for not having it.

### Survey Pilot Testing

The web-based survey was first piloted among 12 volunteers from staff and graduate students and revised for clarity. The second pilot was through the Qualtrics panel to confirm the content validity and reliability before the full launch.

### Statistical Analysis

Descriptive statistics were used to describe the respondents’ demographics, BP telemonitoring behaviors, and health technology behaviors. Categorical variables were reported as frequencies (n [%]), while continuous variables were reported as means and SD. Bivariate analysis using chi-square tests compared patients’ characteristics between RBPM and non-RBPM groups.

The outcome variable was participation in remote BP monitoring (RBPM). Independent variables included demographics, general health status, clinic distance from residence, RBPM awareness, and technology health behaviors. The independent variables used were adopted from factors that have been reported in literature to influence use of digital services [17,19,20,27]

Firth’s [33,34] logistic regression was used to assess the predictors of participation in RBPM. Firth’s logistic regression uses a penalized likelihood approach to account for any separation in the categorical variables due to the small sample size and reduces bias in the parameter estimates. Demographics and technology behaviors were included in the regression model. Age and education interaction variables were also included. Education variables were recoded into 3 categories: less than college, some college, and college graduate or more. Race variables were recoded into two categories: White and other races. Marital status variables were recoded into 3 categories: never married, married, and previously married. Various regression models were fitted using the stepwise forward regression method, and the model with the lowest Akaike information criterion [35] was chosen for the prediction. All analyses were performed using the R Studio software, version 4.2.1 (JJ Allaire and Posit).

## Results

### Description of Participants’ Demographics and Clinical Characteristics

A total of 507 people with hypertension meeting the study criteria were consented to the study and surveyed. The mean age for all participants was 60 (SD 14.7) years (Table 1). The respondents were mostly female (306/507, 60.4%), non-Hispanic (483/507, 95.3%), and White (429/507, 84.6%). Most of the respondents (398/507, 78.5%) lived within 10 miles from their clinics in urban (130/507, 25.6%), and suburban (245/507, 48.3%) areas. More than half reported having had hypertension for 5 years or more (287/507, 56.6%) with the majority reporting their hypertension under control (422/507, 83.2%). Depression or anxiety was the most reported comorbidity (203/507, 40%).

A total of 60 respondents out of the 507 reported participation in RBPM giving a prevalence of 11.8% (Table 1). The RBPM participation group had a significantly lower mean age (46.2, SD 14.7 y) than the non-RBPM participation group (62, SD 13.7 y). The RBPM participation group also had more people in the married category (53.3% vs 39.1%). The majority (75.1%) of those participating in RBPM reported less than 5 years since diagnosis of hypertension compared with 39.1% in the non-RBPM group (Table 1).

**Table 1.** Participants with hypertension demographics and clinical characteristics (cross-sectional survey).

Variable and category		All participants (N=507)	RBPM^a^ participation, n=60 (11.8%)	No RBPM participation, n=447 (88.2%)	*P* value
Age in years, mean (SD)		60.09 (14.7)	46.17 (14.71)	61.96 (13.67)	<.001
**Age groups in years, n (%)**					<.001
	Less than 50	83 (16.4)	32 (53.3)	51 (11.4)	
	50‐74	318 (62.7)	25 (41.7)	293 (65.5)	
	75 and above	106 (20.9)	3 (5)	103 (23)	
**Sex, n (%)**					.30
	Male	201 (39.6)	28 (46.7)	173 (38.7)	
	Female	306 (60.4)	32 (53.3)	274 (61.3)	
**Ethnicity, n (%)**					.09
	Hispanic	24 (4.7)	6 (10)	18 (4)	
	Non-Hispanic	483 (95.3)	54 (90)	429 (96)	
**Race, n (%)**					.03
	American Indian or Alaska Native	4 (0.8)	2 (3.3)	2 (0.4)	
	Asian	7 (1.4)	1 (1.7)	6 (1.3)	
	Black or African American	61 (12)	12 (20)	49 (11)	
	White	429 (84.6)	45 (75.0)	384 (85.9)	
	Other	6 (1.2)	0 (0)	6 (1.3)	
**Education level, n (%)**					.05
	Less than high school	15 (3)	2 (3.3)	13 (2.9)	
	High-school graduate	153 (30.2)	17 (28.3)	136 (30.4)	
	Some college	176 (34.7)	13 (21.7)	163 (36.5)	
	Bachelor’s	148 (29.2)	24 (40)	124 (27.7)	
	Graduate or professional degree	15 (3)	4 (6.7)	11 (2.5)	
**Marital status, n (%)**					.003
	Single	86 (17)	14 (23.3)	72 (16.1)	
	Married	207 (40.8)	32 (53.3)	175 (39.1)	
	Living as married	36 (7.1)	7 (11.7)	29 (6.5)	
	Separated	18 (3.6)	2 (3.3)	16 (3.6)	
	Divorced	94 (18.5)	3 (5)	91 (20.4)	
	Widowed	66 (13)	2 (3.3)	64 (14.3)	
**Annual household income, n (%)**					>.99
	Less than US $20,001	77 (15.2)	9 (15)	68 (15.2)	
	US $ 20 ,001 to $ 35 ,000	120 (23.7)	15 (25)	105 (23.5)	
	US $35 ,001 to $ 50 ,000	94 (18.5)	11 (18.3)	83 (18.6)	
	US $ 50 ,001 to $ 75 ,000	99 (19.5)	11 (18.3)	88 (19.7)	
	US $75,001 or more	106 (20.9)	13 (21.7)	93 (20.8)	
	Prefer not to say	11 (2.2)	1 (1.7)	10 (2.2)	
**Clinic distance, n (%)**					.04
	Less than 5 miles	204 (40.2)	19 (31.7)	185 (41.4)	
	Between 5 and 10 miles	194 (38.3)	32 (53.3)	162 (36.2)	
	More than 10 miles	109 (21.5)	9 (15)	100 (22.4)	
**Area, n (%)**					.01
	Urban	130 (25.6)	24 (40)	106 (23.7)	
	Suburban	245 (48.3)	22 (36.7)	223 (49.9)	
	Exurban	15 (3)	1 (1.7)	14 (3.1)	
	Rural	104 (20.5)	9 (15)	95 (21.3)	
	Blank answer	13 (2.6)	4 (6.7)	9 (2)	
**General health status, n (%)**					.10
	Poor	22 (4.3)	1 (1.7)	21 (4.7)	
	Fair	120 (23.7)	11 (18.3)	109 (24.4)	
	Good	238 (46.9)	26 (43.3)	212 (47.4)	
	Very good	113 (22.3)	18 (30.0)	95 (21.3)	
	Excellent	14 (2.8)	4 (6.7)	10 (2.2)	
**Comorbidity, n (%)**					.01
	Heart condition	0 (0)	0 (0)	0 (0)	
	Diabetes	128 (25.2)	18 (30)	110 (24.6)	
	Depression or anxiety	203 (40)	35 (58.3)	168 (37.6)	
	Chronic kidney disease	24 (4.7)	1 (1.7)	23 (5.1)	
	Other diseases	99 (19.5)	8 (13.3)	91 (20.4)	
	No comorbidity	137 (27)	7 (11.7)	130 (29.1)	
**Hypertension history, n (%)**					<.001
	Less than 1 year	22 (4.3)	0 (0)	22 (4.9)	
	1 year to less than 2 years	44 (8.7)	16 (26.7)	28 (6.3)	
	2 years to less than 3 years	63 (12.4)	15 (25)	48 (10.7)	
	3 years to less than 4 years	47 (9.3)	10 (16.7)	37 (8.3)	
	4 years to less than 5 years	44 (8.7)	4 (6.7)	40 (8.9)	
	5 years or more	287 (56.6)	15 (25.0)	272 (60.9)	
Hypertension medications, mean (SD)		1.61 (0.96)	1.65 (0.73)	1.61 (0.98)	.74
Other medications, mean (SD)		2.92 (2.83)	2.42 (2.32)	2.98 (2.88)	.15
**BP**^**b**^ **under control, n (%)**					.53
	Yes	422 (83.2)	53 (88.3)	369 (82.6)	
	No	46 (9.1)	4 (6.7)	42 (9.4)	
	Don’t know or not sure	39 (7.7)	3 (5.0)	36 (8.1)	
Systolic BP (mm Hg), mean (SD)		131.77 (18.15)	129.24 (23.39)	132.11 (17.34)	.25
Diastolic BP (mm Hg), mean (SD)		80.15 (11.80)	81.64 (13.62)	79.96 (11.555)	.31
**RBPM awareness, n (%)**					<.001
	Yes	165 (32.5)	57 (95.0)	108 (24.2)	
	No	342 (67.5)	3 (5.0)	339 (75.8)	

a RBPM: remote blood pressure monitoring.

b BP: blood pressure.

### BP Self-Monitoring and Telemonitoring Behaviors

Overall, about two-thirds (335/507, 66.1%) of the respondents measured their BP routinely at home with varying frequencies of BP measurement (Multimedia Appendix 1). About 21% (106/507) of all respondents do not measure their BP routinely. Also, 67.5% (342/507) of the respondents were unaware of RBPM and 68.8% (349/507) did not know if their clinic offers RBPM services. Awareness of RBPM was reported in 32.5% (165/507) of the respondents and 34.5% (57/165) of those who were aware reported participation in RBPM (Table 1). Characteristics of those who were aware of RBPM versus those who were unaware are shown in Multimedia Appendix 2. The respondents who were aware of RBPM but not participating in RBPM were older and most of them (65/108, 60.2%) have had hypertension for 5 years or more compared with those participating in RBPM (Multimedia Appendix 3). Taking BP medications as prescribed by the health care provider was the most common self-BP control behavior among all respondents.

Most respondents in the RBPM group measure their BP at home (55/60, 91.7%) and 61.7% (37/60) engage in daily BP measurement compared with 62.6% (280/447) and 25.1% (112/447), respectively, among the non-RBPM group (Multimedia Appendix 1). A greater number of those in the RBPM group reported tracking their BP measurements with mHealth (37/60, 61.7%) than those in the non-RBPM group (70/447, 15.6%).

Among those participating in RBPM, the most reported RBPM provider was doctors, while RBPM frequency was mostly daily and several times per week (Multimedia Appendix 4). The electronic health records or patient portal was the most common channel of RBPM communication between the respondents and their health care providers. The feedback message to the respondents were mainly acknowledgement of BP measurement receipt and interpretation of measurement as low, high, or normal (Multimedia Appendix 4).

The top 2 reasons for not participating in RBPM were that doctors have yet to ask them to participate and their lack of awareness of RBPM (Multimedia Appendix 5). About three-quarters of those not participating in RBPM reported that they would likely participate in RBPM if offered.

### Technology Health Behaviors

Most of the 507 respondents reported having a smartphone (469/507, 92.5%), tablet computer (323/507, 63.7%), laptop or desktop computer (440/507, 86.8%), health-related apps (299/507, 59%), and home BP monitoring device (399/507, 78.7%; Multimedia Appendix 6). About three-quarters of the respondents have communicated with their doctor or doctor’s office through emails (377/507, 74.4%) or the internet and checked their medical test results electronically (381/507, 75.1%). About half reported that they made health decisions (277/507, 54.6%) and achieved health goals (235/507, 46.4%) with mHealth, and shared health information electronically with their health care providers (252/507, 49.7%; Multimedia Appendix 7).

The ownership of tablet computers and health-related apps was significantly higher among the RBPM group than the non-RBPM groups (85% vs 60.9%; 93.3% vs 54.4%; *P*<.001 respectively). The RBPM group also had a significantly higher proportion of people who own and use home BP monitoring devices than the non-RBPM group (96.7% vs 64.7%, *P*<.001). All the technology use health behaviors were observed more significantly in the RBPM group than the non-RBPM group (Multimedia Appendix 7).

Among the 108 respondents who reported not having a home BP device, the top reported reasons for not owning a home BP monitoring device included dependence on a health provider for BP measurement (46/108, 42.6%), not being able to afford one (38/108, 35.2%), feeling that BP is under control (23/108, 21.3%), thinking that they do not need the device (20/108, 18.5%), and not having seen a device that works well for them (20/108,18.5%).

**Figure 1.**
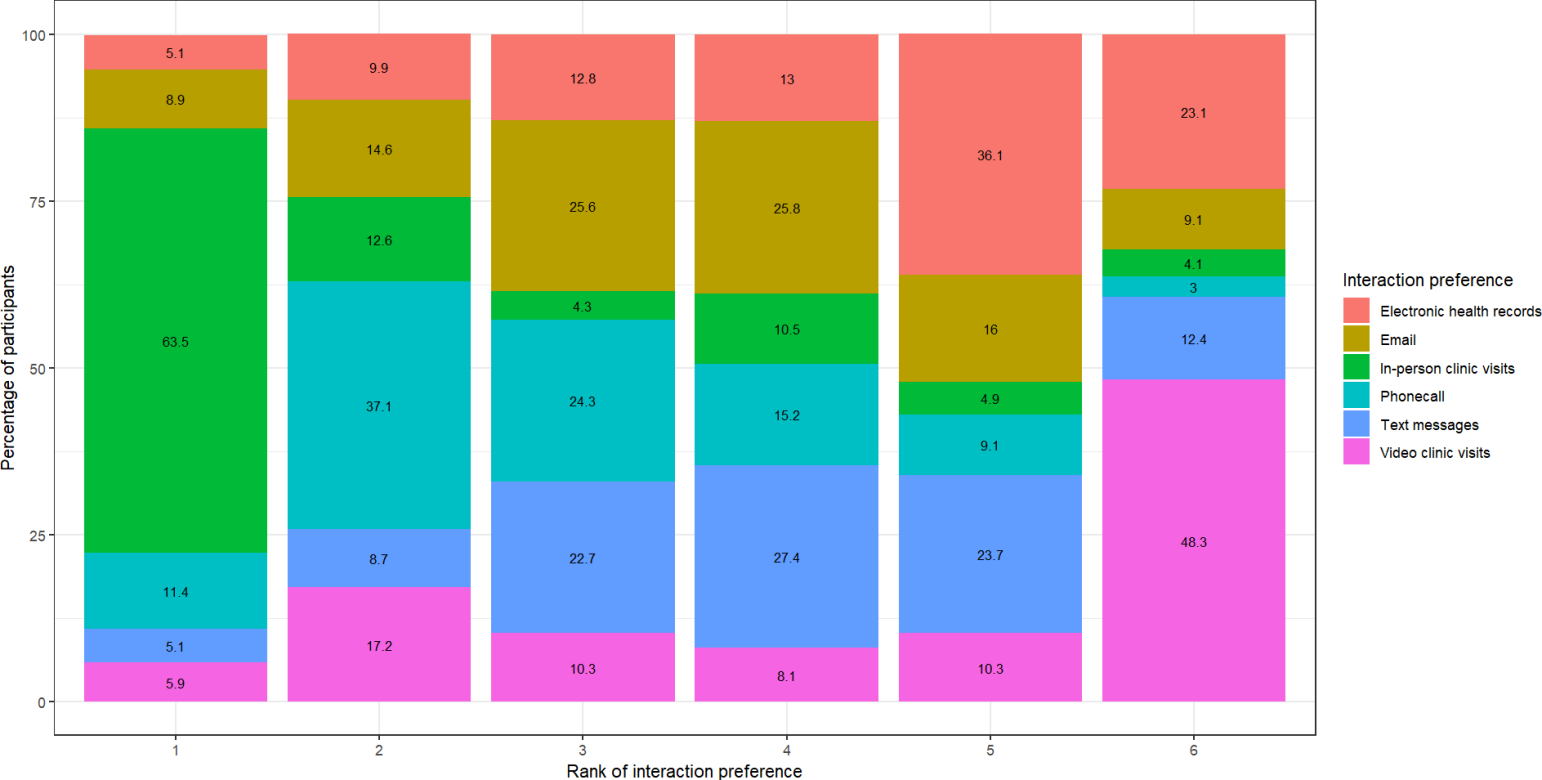
Ranking of participants’ interaction preferences with health care providers regarding their hypertension management among adults with hypertension (cross-sectional survey; 1 denotes the most preferred and 6 is the least preferred).

Figure 1 shows the patients’ interaction preferences with their health care provider regarding their BP management. Overall, the most preferred mode of patient-provider interaction was in-person clinic visits, while the least preferred was video visits. Among the electronic communication methods, phone calls were the most preferred, followed by email, then SMS text messages. Video visits remained the least preferred interaction method. There was no difference in the order of interaction preferences across age and educational groups. The order of preferences for patient-provider interaction channels remained the same in the RBPM and non-RBPM groups as in the overall participants.

### Predictors of RBPM Participation

Overall, the statistically significant predictors of participation in RBPM were awareness of RBPM (adjusted odds ratio [AOR] 34.65, 95% CI 11.35‐150.31; *P*<.001) and sharing electronic health information with a health care provider (AOR 4.90, 95% CI 1.39‐21.64; *P*=.01) (Table 2). Age, education level, marital status, clinic distance, ownership of technology, and other behavioral variables were not statistically significant predictors. Sharing electronic health information with a health care provider was the only significant predictor (AOR 6.99, 95% CI 1.62‐47.44; *P*=.007) of RBPM participation among those who were aware of RBPM (Multimedia Appendix 8).

**Table 2.** Predictors of RBPM^a^ participation using Firth’s logistic regression among adults with hypertension (cross-sectional survey).

Predictor variables and categories		Adjusted odds ratio (95% CI)	*P* values
Age		0.99 (0.94-1.06)	.97
**Sex** ^b^			
	Male	0.80 (0.32-1.96)	.63
**Education level** ^c^			
	Some college	0.69 (0.01-34.93)	.86
	College graduate or more	24.81 (0.38-1938.02)	.13
**Race** ^d^			
	American Indian or Alaska Native or Asian or Black or African American or other races	0.62 (0.21-1.71)	.36
**Marital status** ^e^			
	Married	1.33 (0.45-4.10)	.61
	Previously married	0.53 (0.12-2.17)	.38
**Clinic distance from residence** ^f^			
	Between 5 and 10 miles	1.42 (0.55-3.73)	.47
	More than 10 miles	0.64 (0.18-2.20)	.48
**RBPM awareness** ^g^			
	Yes	34.65 (11.35-150.31)	<.001
**BP^h^ under control** ^g^			
	Yes	0.32 (0.06-1.78)	.18
	Don’t know or unsure	0.23 (0.02-2.78)	.25
**Have tablet** ^g^			
	Yes	1.14 (0.40-3.38)	.81
**Have smartphone** ^g^			
	Yes	0.86 (0.05-24.04)	.92
**Have basic cellphone only** ^g^			
	Yes	2.30 (0.79-6.87)	.13
**Have computer** ^g^			
	Yes	0.81 (0.23-3.17)	.25
**Have health apps** ^g^			
	Yes	2.31 (0.58-12.03)	.24
**Electronic communication with doctor or doctor’s office via email or internet** ^g^			
	Yes	1.11 (0.19-9.57)	.91
**Sent or received SMS text message from doctor** ^g^			
	Yes	1.90 (0.63-6.38)	.26
**Shared health information from electronic device, tablet, or smartphone with health provider** ^g^			
	Yes	4.90 (1.39-21.64)	.01
**Made health decision with mobile health** ^g^			
	Yes	0.34 (0.08-1.26)	.11
**Achieved health goals with mobile health** ^g^			
	Yes	2.55 (0.78-9.15)	.12
**Have checked medical test results electronically** ^**g**^			
	Yes	0.36 (0.08-1.48)	.16
**Time since hypertension diagnosis** ^**i**^			
	Less than 1 year	0.29 (0.002-5.29)	.46
	1 year - <2 years	1.80 (0.40-7.93)	.44
	2 years - <3 years	2.02 (0.57-7.10)	.27
	3 years - <4 years	0.86 (0.19-3.54)	.83
	4 years - <5 years	0.55 (0.11-2.31)	.43
**Age and education interaction** ^**j**^			
	Age and some college education	0.98 (0.91-1.05)	.58
	Age and college graduate or more	0.92 (0.85-1.00)	.47

a RBPM: remote blood pressure monitoring.

b Reference: female sex.

c Reference: less than college education.

d Reference: White race.

e Reference: never married.

f Reference: clinic distance less than 5 miles from residence.

g Reference: no response.

h BP: blood pressure.

i Reference: 5 years or more.

j Reference: age and less than college education.

## Discussion

### Principal Findings

We found that more than three-quarters of patients with hypertension were not participating in RBPM. Nonparticipation in RBPM was mostly due to not being asked to participate by their health care providers and lack of awareness of RBPM, as only one-third of the patients were aware of RBPM. These findings show health care providers’ important role in helping patients take up health-improving strategies. A doctor’s referral or recommendation has been identified as influential in a patient’s telemedicine utilization [36]. A related study on the utilization of teleconsultation among adult epileptic patients in a low-income setting showed that only about 32% had used teleconsultation and more than half (58%) of the patients were not aware of teleconsultation services [37]. Patients will likely take up health-improving programs like RBPM if they know the program and its benefits. A review of the impact of telemedicine during the COVID-19 pandemic by Omboni et al [38], identified patients’ awareness as an important factor in the uptake of digital health services. This finding is also supported by the high number (74.9%) of non-RBPM patients in our study who reported they would likely participate in RBPM if offered. Another study [27] assessing willingness to take up telemonitoring programs among patients with diabetes and/or hypertension found that more than half (52.5%) of the respondents were willing to take up telemonitoring programs. Despite the differences in proportions of patients willing to participate in BP telemonitoring in these two studies, it shows that a sizable number would likely participate had it been brought to their awareness or if they received a recommendation. A concerted effort among all health care providers (physicians, nurses, pharmacists, etc) toward RBPM recommendations to their patients could provide the encouragement and support needed to get more patients with hypertension to participate in RBPM. However, the recommendation of the RBPM program depends on its availability and accessibility in health care institutions [5,24]. It therefore calls for the provision of the technical infrastructure (eg, adequate RBPM system and home BP monitoring device for patients) and appropriate reimbursements to aid health care providers in rendering RBPM services.

Our study showed that phone calls were the most preferred electronic communication method regardless of participation or nonparticipation in RBPM. This could be because of the simplicity of making and answering calls as easy-to-use technology is a required feature of digital services [13,39]. It could also be because over 80% of our participants were 50 years and above. Older patients have been found to prefer phone call interactions over other electronic modes of communication [40-42]. It is important for health care providers or office staff to identify the patients’ electronic communication preferences regarding their health management [43]. The reimbursement of phone-based care during the COVID-19 pandemic greatly improved patient care [44]. It is therefore quite unfortunate that reimbursement of this health improvement policy will end by December 31, 2024, according to the United States Consolidated Appropriation Act, 2023 [45]. Given the health benefits of phone-based health management, health care policymakers should rather make it permanent and extend similar reimbursement to RBPM services to help health care providers improve their patients’ health. Engaging patients electronically through their preferred medium would likely promote adherence to health management protocols.

In addition to awareness, sharing health information from electronic devices with health care providers was positively associated with RBPM participation in the adjusted analyses. This previous technology-related health behavior shows that experience with an action makes it more likely to engage in a similar action. This finding is supported by a study among patients with hypertension where prior communication with the doctor or doctor’s office through email or the internet was a significant predictor of communicating with the doctor via SMS text messaging [20]. Health care institutions should consider making electronic communications channels such as SMS texting, emails, and so on, available and accessible for their patients. Measures to improve awareness of RBPM among patients with hypertension as well as encourage use of electronic health devices and sharing of their health information may increase participation in RBPM.

RBPM is a valuable way of engaging patients with hypertension in their disease management to achieve BP control and mitigate the consequences of uncontrolled BP. However, more must be done to get patients to embrace this digital service option. The availability of secure RBPM infrastructure accessible to patients is essential. Proactive actions like building RBPM into routine health care and ensuring that every patient diagnosed with hypertension has access to it could go a long way in increasing participation. Reimbursement of RBPM services and insurance coverage of home BP monitoring devices could help increase RBPM accessibility to patients. It may be helpful for health systems to spread the awareness and benefits of RBPM to the general population of patients with hypertension but start the RBPM service with the most severe cases and gradually expand it to all patients according to their needs.

### Study Limitations

This study is limited by its cross-sectional design, and the generalizability may be limited because the sample included mostly non-Hispanic White patients with hypertension living mainly in urban and suburban areas. Over 80% of our sample reported having their BP under control, and most (72%) reported being in good health. Therefore, a more diverse population based on race, ethnicity, residential area, BP control status, and health status is warranted for future studies. A self-administered electronic survey may have excluded those not technology savvy from participating and introduced bias in the responses. As the study is related to technology use, respondents could be overly positive or supportive of the RBPM. The study was also limited to people with hypertension who were enrolled in the Qualtrics panel. However, our study is strengthened by having a large sample size to make predictions. We also recruited representatives from all age brackets and educational levels to mitigate age and education biases.

### Conclusion

These results suggest low RBPM participation among patients with hypertension in the United States. Creating awareness of RBPM and encouraging patients to share their health information electronically with their health care providers may increase RBPM participation. This calls for health care policies ensuring RBPM availability, accessibility, and service reimbursement in our health care systems.

## Supplementary material

10.2196/56954 Multimedia Appendix 1 Self–blood pressure monitoring behaviors.

10.2196/56954 Multimedia Appendix 2 Respondents’ characteristics stratified by remote blood pressure monitoring awareness.

10.2196/56954 Multimedia Appendix 3 Aware but not participating in remote blood pressure monitoring.

10.2196/56954 Multimedia Appendix 4 Remote blood pressure monitoring (RBPM) strategies.

10.2196/56954 Multimedia Appendix 5 Reasons for not participating and likelihood of participating in remote blood pressure monitoring.

10.2196/56954 Multimedia Appendix 6 Technology ownership.

10.2196/56954 Multimedia Appendix 7 Technology use.

10.2196/56954 Multimedia Appendix 8 Predictors of remote blood pressure monitoring participation among those who are aware.

10.2196/56954 Checklist 1 Checklist for Reporting Results of Internet E-Surveys (CHERRIES).

## Abbreviations

AOR: adjusted odds ratio
BP: blood pressure
CHERRIES: Checklist for Reporting Results of Internet E-Surveys
mHealth: mobile health
RBPM: remote blood pressure monitoring
