# Supplementary material for: Behavioral Factors Related to Participation in Remote Blood Pressure Monitoring Among Adults With Hypertension: Cross-Sectional Study
Source: JMIR Form Res. 2024 Dec 23;8:e56954. doi: 10.2196/56954 (PMC11684531; doi:10.2196/56954)
Supplement: Multimedia Appendix 1 [file formative-v8-e56954-s001.docx]

Appendix 1. Self BP monitoring behaviors

| Variable | Category | All Participants  N = 507 | RBPM Participation  n= 60 (11.8%) | No RBPM Participation  n= 447 (88.2%) | P-value |
| --- | --- | --- | --- | --- | --- |
| Routine BP measurement venue |  |  |  |  | <0.001 |
|  | At home | 335 (66.1) | 55 (91.7) | 280 (62.6) |  |
|  | At the pharmacy | 83 (16.4) | 16 (26.7) | 67 (15.0) |  |
|  | At the clinic | 109 (21.5) | 18 (30.0) | 91 (20.4) |  |
|  | At work | 20 (3.9) | 12 (20.0) | 8 (1.8) |  |
|  | Some other places | 6 (1.2) | 1 (1.7) | 5 (1.1) |  |
|  | Do not measure BP routinely | 106 (20.9) | 0 (0.0) | 106 (23.7) |  |
| Frequency of Home BP measurement |  |  |  |  | <0.001 |
|  | Daily | 149 (29.4) | 37 (61.7) | 112 (25.1) |  |
|  | Several times a week | 108 (21.3) | 19 (31.7) | 89 (19.9) |  |
|  | Once a week | 33 (6.5) | 1 (1.7) | 32 (7.2) |  |
|  | 1 to 3 times a month | 46 (9.1) | 1 (1.7) | 45 (10.1) |  |
|  | Once in 3 months | 9 (1.8) | 0 (0.0) | 9 (2.0) |  |
|  | Once in 6 months | 2 (0.4) | 0 (0.0) | 2 (0.4) |  |
| BP tracking strategy |  |  |  |  | <0.001 |
|  | Writing on paper | 163 (32.1) | 19 (31.7) | 144 (32.2) |  |
|  | Writing on calendar | 35 (6.9) | 12 (20.0) | 23 (5.1) |  |
|  | Writing on App on phone/tablet/computer | 59 (11.6) | 24 (40.0) | 35 (7.8) |  |
|  | Writing on Excel sheet or Notepad on phone/tablet/computer | 13 (2.6) | 1 (1.7) | 12 (2.7) |  |
|  | Do not keep track | 51 (10.1) | 1 (1.7) | 50 (11.2) |  |
|  | Other strategies | 26 (5.1) | 1 (1.7) | 25 (5.6) |  |
| How Self- measured BP is shared with health provider |  |  |  |  | <0.001 |
|  | By taking them to doctor visits | 235 (46.4) | 33 (55.0) | 202 (45.2) |  |
|  | By device automatic transfer to doctor | 19 (3.7) | 15 (25.0) | 4 (0.9) |  |
|  | By email to doctor | 19 (3.7) | 12 (20.0) | 7 (1.6) |  |
|  | By electronic health record/patient portal to doctor | 9 (1.8) | 4 (6.7) | 5 (1.1) |  |
|  | By text messages to doctor | 2 (0.4) | 0 (0.0) | 2 (0.4) |  |
|  | Do not share with health provider | 77 (15.2) | 2 (3.3) | 75 (16.8) |  |
| RBPM awareness |  |  |  |  | ˂0.001 |
|  | Yes | 165 (32.5) | 57 (95.0) | 108 (24.2) |  |
|  | No | 342(67.5) | 3 (5.0) | 339 (75.8) |  |
| RBPM offered in clinic |  |  |  |  | ˂0.001 |
|  | Yes | 66 (13.0) | 57 (95.0) | 9 (2.0) |  |
|  | No | 92 (18.1) | 0 (0.0) | 92 (20.6) |  |
|  | Don’t know | 349 (68.8) | 3 (5.0) | 346 (77.4) |  |

RBPM: Remote blood pressure monitoring, BP: Blood pressure
