# Supplementary material for: Behavioral Factors Related to Participation in Remote Blood Pressure Monitoring Among Adults With Hypertension: Cross-Sectional Study
Source: JMIR Form Res. 2024 Dec 23;8:e56954. doi: 10.2196/56954 (PMC11684531; doi:10.2196/56954)
Supplement: Multimedia Appendix 2 [file formative-v8-e56954-s002.docx]

Appendix 2: Respondents’ characteristics stratified by RBPM Awareness

| Variable | Category | All Participants  N = 507 | RBPM Awareness  n= 165 (32.5%) | No RBPM Awareness  n= 342 (67.5%) | P-value |
| --- | --- | --- | --- | --- | --- |
| **PARTICIPANTS’ DEMOGRAPHICS** | | | | | |
| Age (mean, SD) |  | 60.09 (14.7) | 55.97 (15.96) | 62.08 (13.63) | ˂0.001 |
| Age groups |  |  |  |  | ˂0.001 |
|  | Less than 50 | 83 (16.4) | 45 (27.3) | 38 (11.1) |  |
|  | 50-74 | 318 (62.7) | 95 (57.6) | 223 (65.2) |  |
|  | 75 and above | 106 (20.9) | 25 (15.2) | 81 (23.7) |  |
| Gender |  |  |  |  | 0.006 |
|  | Male | 201 (39.6) | 80 (48.5) | 121 (35.4) |  |
|  | Female | 306 (60.4) | 85 (51.5) | 221 (64.6) |  |
| Ethnicity |  |  |  |  | 0.100 |
|  | Hispanic | 24 (4.7) | 12 (7.3) | 12 (3.5) |  |
|  | Non-Hispanic | 483 (95.3) | 153 (92.7) | 330 (96.5) |  |
| Race |  |  |  |  | 0.045 |
|  | American Indian or Alaska native | 4 (0.8)) | 3 (1.8) | 1 (0.3) |  |
|  | Asian | 7 (1.4) | 3 (1.8) | 4 (1.2) |  |
|  | Black or African American | 61 (12.0) | 25 (15.2) | 36 (10.5) |  |
|  | White | 429 (84.6) | 130 (78.8) | 299 (87.4) |  |
|  | Other | 6 (1.2) | 4 (2.4) | 2 (0.6) |  |
| Education level |  |  |  |  | ˂0.001 |
|  | Less than High Sch | 15 (3.0) | 4 (2.4) | 11 (3.2) |  |
|  | High Sch Graduate | 153 (30.2) | 31 (18.8) | 122 (35.7) |  |
|  | Some College | 176 (34.7) | 63 (38.2) | 113 (33.0) |  |
|  | Bachelor’s | 148 (29.2) | 57 (34.5) | 91 (26.6) |  |
|  | Graduate and /or Prof degree | 15 (3.0) | 10 (6.1) | 5 (1.5) |  |
| Marital Status |  |  |  |  | 0.063 |
|  | Single | 86 (17.0) | 32 (19.4) | 54 (15.8) |  |
|  | Married | 207 (40.8) | 79 (47.9) | 128 (37.4) |  |
|  | Living as married | 36 (7.1) | 10 (6.1) | 26 (7.6) |  |
|  | Separated | 18 (3.6) | 6 (3.6) | 12 (3.5) |  |
|  | Divorced | 94 (18.5) | 20 (12.1) | 74 (21.6) |  |
|  | Widowed | 66 (13.0) | 18 (10.9) | 48 (14.0) |  |
| Annual household income |  |  |  |  | 0.362 |
|  | Less than $20,001 | 77 (15.2) | 21 (12.7) | 56 (16.4) |  |
|  | $20,001 to $35,000 | 120 (23.7) | 34 (20.6) | 86 (25.1) |  |
|  | 35,001 to $50,000 | 94 (18.5) | 32 (19.4) | 62 (18.1) |  |
|  | $50,001 to $75,000 | 99 (19.5) | 39 (23.6) | 60 (17.5) |  |
|  | $75,001 or more | 106 (20.9) | 37 (22.4) | 69 (20.2) |  |
|  | Prefer not to say | 11 (2.2) | 2 (1.2) | 9 (2.6) |  |
| Clinic Distance |  |  |  |  | 0.025 |
|  | Less than 5 miles | 204 (40.2) | 53 (32.1) | 151 (44.2) |  |
|  | Between 5 and 10 miles | 194 (38.3) | 75 (45.5) | 119 (34.8) |  |
|  | More than 10 miles | 109 (21.5) | 37 (22.4) | 72 (21.1) |  |
| Area |  |  |  |  | 0.738 |
|  | Urban | 130 (25.6) | 48 (29.1) | 82 (24.0) |  |
|  | Suburban | 245 (48.3) | 74 (44.8) | 171 (50.0) |  |
|  | Exurban | 15 (3.0) | 5 (3.0) | 10 (2.9) |  |
|  | Rural | 104 (20.5) | 33 (20.0) | 71 (20.8) |  |
|  | Blank answer | 13 (2.6) | 5 (3.0) | 8 (2.3) |  |
|  |  |  |  |  |  |
| **CLINICAL CHARACTERISTICS** | | | | | |
| General Health Status |  |  |  |  | 0.004 |
|  | Poor | 22 (4.3) | 4 (2.4) | 18 (5.3) |  |
|  | Fair | 120 (23.7) | 34 (20.6) | 86 (25.1) |  |
|  | Good | 238 (46.9) | 70 (42.4) | 168 (49.1) |  |
|  | Very good | 113 (22.3) | 48 (29.1) | 65 (19.0) |  |
|  | Excellent | 14 (2.8) | 9 (5.5) | 5 (1.5) |  |
| Comorbidity |  |  |  |  | 0.588 |
|  | Heart Condition | 0 (0.0) | 0 (0.0) | 0 (0.0) |  |
|  | Diabetes | 128 (25.2) | 41 (24.8) | 87 (25.4) |  |
|  | Depression or Anxiety | 203 (40.0) | 67 (40.6) | 136 (39.8) |  |
|  | Chronic kidney disease | 24 (4.7) | 7 (4.2) | 17 (5.0) |  |
|  | Other diseases | 99 (19.5) | 33 (20.0) | 66 (19.3) |  |
|  | No comorbidity | 137 (27.0) | 39 (23.6) | 98 (28.7) |  |
| HTN History |  |  |  |  | 0.008 |
|  | Less than 1 year | 22 (4.3) | 3 (1.8) | 19 (5.6) |  |
|  | 1 year – less than 2 years | 44 (8.7) | 22 (13.3) | 22 (6.4) |  |
|  | 2 years – less than 3 years | 63 (12.4) | 23 (13.9) | 40 (11.7) |  |
|  | 3 years – less than 4 years | 47 (9.3) | 19 (11.5) | 28 (8.2) |  |
|  | 4 years – less than 5 years | 44 (8.7) | 18 (10.9) | 26 (7.6) |  |
|  | 5 years or more | 287 (56.6) | 80 (48.5) | 207 (60.5) |  |
| HTN Meds (mean, SD) |  | 1.61 (0.96) | 1.61 (0.75) | 1.61 (1.04) | 0.991 |
| Other Meds (mean, SD) |  | 2.92 (2.83) | 2.58 (3.00) | 3.08 (2.73) | 0.065 |
| BP under control |  |  |  |  | 0.047 |
|  | Yes | 422 (83.2) | 147 (89.1) | 275 (80.4) |  |
|  | No | 46 (9.1) | 9 (5.5) | 37 (10.8) |  |
|  | Don’t know or Not sure | 39 (7.7) | 9 (5.5) | 30 (8.8) |  |
| Systolic BP (mean, SD) |  | 131.77 (18.15) | 129.20 (18.79) | 133.01 (17.73) | 0.027 |
| Diastolic BP (mean, SD) |  | 80.15 (11.80) | 79.13 (11.37) | 80.64 (11.99) | 0.180 |
|  |  |  |  |  |  |
| **BP MONITORING BEHAVIORS** | | | | | |
| Routine BP measurement venue |  |  |  |  | <0.001 |
|  | At home | 335 (66.1) | 131 (79.4) | 204 (59.6) |  |
|  | At the pharmacy | 83 (16.4) | 37 (22.4) | 46 (13.5) |  |
|  | At the clinic | 109 (21.5) | 39 (23.6) | 70 (20.5) |  |
|  | At work | 20 (3.9) | 15 (9.1) | 5 (1.5) |  |
|  | Some other places | 6 (1.2) | 2 (1.2) | 4 (1.2) |  |
|  | Do not measure BP routinely | 106 (20.9) | 18 (10.9) | 88 (25.7) |  |
| Frequency of Home BP measurement |  |  |  |  | <0.001 |
|  | Daily | 149 (29.4) | 69 (41.8) | 80 (23.4) |  |
|  | Several times a week | 108 (21.3) | 41 (24.8) | 67 (19.6) |  |
|  | Once a week | 33 (6.5) | 10 (6.1) | 23 (6.7) |  |
|  | 1 to 3 times a month | 46 (9.1) | 10 (6.1) | 36 (10.5) |  |
|  | Once in 3 months | 9 (1.8) | 3 (1.8) | 6 (1.8) |  |
|  | Once in 6 months | 2 (0.4) | 0 (0.0) | 2 (0.6) |  |
| BP tracking strategy |  |  |  |  | <0.001 |
|  | Writing on paper | 163 (32.1) | 51 (30.9) | 112 (32.7) |  |
|  | Writing on calendar | 35 (6.9) | 19 (11.5) | 16 (4.7) |  |
|  | Writing on App on phone/tablet/computer | 59 (11.6) | 34 (20.6) | 25 (7.3) |  |
|  | Writing on Excel sheet or Notepad on phone/tablet/computer | 13 (2.6) | 6 (3.6) | 7 (2.0) |  |
|  | Do not keep track | 51 (10.1) | 13 (7.9) | 38 (11.1) |  |
|  | Other strategies | 26 (5.1) | 10 (6.1) | 16 (4.7) |  |
| How Self- measured BP is shared with health provider |  |  |  |  | <0.001 |
|  | By taking them to doctor visits | 235 (46.4) | 93 (56.4) | 142 (41.5) |  |
|  | By device automatic transfer to doctor | 19 (3.7) | 17 (10.3) | 2 (0.6) |  |
|  | By email to doctor | 19 (3.7) | 12 (7.3) | 7 (2.0) |  |
|  | By electronic health record/patient portal to doctor | 9 (1.8) | 6 (3.6) | 3 (0.9) |  |
|  | By text messages to doctor | 2 (0.4) | 1 (0.6) | 1 (0.3) |  |
|  | Do not share with health provider | 77 (15.2) | 15 (9.1) | 62 (18.1) |  |
| RBPM participation |  |  |  |  | ˂0.001 |
|  | Yes | 60 (11.8) | 57 (34.5) | 3 (0.9) |  |
|  | No | 447 (88.2) | 108 (65.5) | 339 (99.1) |  |
| RBPM offered in clinic |  |  |  |  | ˂0.001 |
|  | Yes | 66 (13.0) | 65 (39.4) | 1 (0.3) |  |
|  | No | 92 (18.1) | 30 (18.2) | 62 (18.1) |  |
|  | Don’t know | 349 (68.8) | 70 (42.4) | 279 (81.6) |  |
| **BP CONTROL BEHAVIORS** | | | | | |
| Behaviors |  |  |  |  |  |
|  | Taking BP meds as prescribed | 448 (88.4) | 150 (90.9) | 298 (87.1) |  |
|  | Exercise | 243 (47.9) | 91 (55.2) | 152 (44.4) |  |
|  | Low sodium diet | 216 (42.6) | 80 (48.5) | 136 (39.8) |  |
|  | Low carbohydrate diet | 80 (15.8) | 32 (19.4) | 48 (14.0) |  |
|  | Adequate hydration with lots of water | 204 (40.2) | 64 (38.8) | 140 (40.9) |  |
|  | Adequate sleep | 195 (38.5) | 75 (45.5) | 120 (35.1) |  |
|  | Reduction in coffee intake | 88 (17.4) | 30 (18.2) | 58 (17.0) |  |
|  | Meditation | 58 (11.4) | 28 (17.0) | 30 (8.8) |  |
|  | Breathing exercises | 70 (13.8) | 28 (17.0) | 42 (12.3) |  |
|  | Stress reduction | 117 (23.1) | 55 (33.3) | 62 (18.1) |  |
|  | Reducing alcohol consumption | 98 (19.3) | 35 (21.2) | 63 (18.4) |  |
|  | Periodic health checks | 211 (41.6) | 63 (38.2) | 148 (43.3) |  |
|  | None of the above | 0 (0.0) | 0 (0.0) | 0 (0.0) |  |
|  | Other behaviors not listed | 3 (0.6) | 0 (0.0) | 3 (0.9) |  |
| **TECHNOLOGY OWNERSHIP** | | | | | |
| Have Basic cellphone only |  |  |  |  | 0.528 |
|  | Yes | 89 (17.6) | 32 (19.4) | 57 (16.7) |  |
|  | No | 418 (82.4) | 133 (80.6) | 285 (83.3) |  |
| Have Smartphone |  |  |  |  | 0.302 |
|  | Yes | 469 (92.5) | 156 (94.5) | 313 (91.5) |  |
|  | No | 38 (7.5) | 9 (5.5) | 29 (8.5) |  |
| Have Tablet |  |  |  |  | 0.041 |
|  | Yes | 323 (63.7) | 116 (70.3) | 207 (60.5) |  |
|  | No | 184 (36.3) | 49 (29.7) | 135 (39.5) |  |
| Have Desktop or Laptop |  |  |  |  | 0.932 |
|  | Yes | 440 (86.8) | 144 (87.3) | 296 (86.5) |  |
|  | No | 67 (13.2) | 21 (12.7) | 46 (13.5) |  |
| Have home BP device |  |  |  |  | ˂0.001 |
|  | Yes, I use it | 347 (68.4) | 133 (80.6) | 214 (62.6) |  |
|  | Yes, don’t use it | 52 (10.3) | 10 (6.1) | 42 (12.3) |  |
|  | No | 108 (21.3) | 22 (13.3) | 86 (25.1) |  |
| Home BP device payment |  |  |  |  | 0.002 |
|  | Paid by self | 286 (56.4) | 98 (59.4) | 188 (55.0) |  |
|  | Gifted | 25 (4.9) | 6 (3.6) | 19 (5.6) |  |
|  | Insurance paid | 78 (15.4) | 37 (22.4) | 41 (12.0) |  |
|  | Other | 10 (2.0) | 2 (1.2) | 8 (2.3) |  |
|  | No Home BP device | 108 (21.3) | 22 (13.3) | 86 (25.1) |  |
| Have Health Apps |  |  |  |  | ˂0.001 |
|  | Yes | 299 (59.0) | 121 (73.3) | 178 (52.0) |  |
|  | No | 208 (41.0) | 44 (26.7) | 164 (48.0) |  |
| **TECHNOLOGY USE** | | | | | |
| Electronic communication with doctor or doctor’s office via email or internet |  |  |  |  | ˂0.001 |
|  | Yes | 377 (74.4) | 142 (86.1) | 235 (68.7) |  |
|  | No | 130 (25.6) | 23 (13.9) | 107 (31.3) |  |
| Electronic checking of medical tests |  |  |  |  | 0.062 |
|  | Yes | 381 (75.1) | 133 (80.6) | 248 (72.5) |  |
|  | No | 126 (24.9) | 32 (19.4) | 94 (27.5) |  |
| Achieving health goals with mHealth |  |  |  |  | ˂0.001 |
|  | Yes | 235 (46.4) | 99 (60.0) | 136 (39.8) |  |
|  | No | 272 (53.6) | 66 (40.0) | 206 (60.2) |  |
| Health decision making with mHealth |  |  |  |  | ˂0.001 |
|  | Yes | 277 (54.6) | 118 (71.5) | 159 (46.5) |  |
|  | No | 230 (45.4) | 47 (28.5) | 183 (53.5) |  |
| mHealth helps discussion with health care provider |  |  |  |  | ˂0.001 |
|  | Yes | 305 (60.2) | 132 (80.0) | 173 (50.6) |  |
|  | No | 202 (39.8) | 33 (20.0) | 169 (49.4) |  |
| Shared health information electronically with health care provider |  |  |  |  | ˂0.001 |
|  | Yes | 252 (49.7) | 111 (67.3) | 141 (41.2) |  |
|  | No | 255 (50.3) | 54 (32.7) | 201 (58.8) |  |
| Text messaging with doctor |  |  |  |  | ˂0.001 |
|  | Yes | 311 (61.3) | 121 (73.3) | 190 (55.6) |  |
|  | No | 196 (38.7) | 44 (26.7) | 152 (44.4) |  |
|  |  |  |  |  |  |
