# Supplementary material for: Behavioral Factors Related to Participation in Remote Blood Pressure Monitoring Among Adults With Hypertension: Cross-Sectional Study
Source: JMIR Form Res. 2024 Dec 23;8:e56954. doi: 10.2196/56954 (PMC11684531; doi:10.2196/56954)
Supplement: Multimedia Appendix 3 [file formative-v8-e56954-s003.docx]

Appendix 3: Characteristics of those who are aware of RBPM but are not participating in RBPM.

| Variable | Category | All Participants  N = 507 | RBPM Participation  n= 60 (11.8%) | RBPM Aware but No RBPM Participation  n= 108 (65.5%) |
| --- | --- | --- | --- | --- |
|  |  |  |  |  |
| **Age in years (mean, SD)** |  | 60.09 (14.7) | 46.17 (14.71) | 61.34 (13.72) |
| **Age groups in years** |  |  |  |  |
|  | Less than 50 | 83 (16.4) | 32 (53.3) | 13 (12.0) |
|  | 50-74 | 318 (62.7) | 25 (41.7) | 73 (67.6) |
|  | 75 and above | 106 (20.9) | 3 (5.0) | 22 (20.4) |
| **Sex** |  |  |  |  |
|  | Male | 201 (39.6) | 28 (46.7) | 52 (48.1) |
|  | Female | 306 (60.4) | 32 (53.3) | 56 (51.9) |
| **Ethnicity** |  |  |  |  |
|  | Hispanic | 24 (4.7) | 6 (10.0) | 6 (5.6) |
|  | Non-Hispanic | 483 (95.3) | 54 (90.0) | 102 (94.4) |
| **Race** |  |  |  |  |
|  | American Indian or Alaska Native | 4 (0.8)) | 2 (3.3) | 1 (0.9) |
|  | Asian | 7 (1.4) | 1 (1.7) | 2 (1.9) |
|  | Black or African American | 61 (12.0) | 12 (20.0) | 13 (12.0) |
|  | White | 429 (84.6) | 45 (75.0) | 88 (81.5) |
|  | Other | 6 (1.2) | 0 (0.0) | 4 (3.7) |
| **Education level** |  |  |  |  |
|  | Less than High School | 15 (3.0) | 2 (3.3) | 2 (1.9) |
|  | High School Graduate | 153 (30.2) | 17 (28.3) | 16 (14.8) |
|  | Some College | 176 (34.7) | 13 (21.7) | 50 (46.3) |
|  | Bachelor’s | 148 (29.2) | 24 (40.0) | 34 (31.5) |
|  | Graduate and /or Prof degree | 15 (3.0) | 4 (6.7) | 6 (5.6) |
| **Marital Status** |  |  |  |  |
|  | Single | 86 (17.0) | 14 (23.3) | 18 (16.7) |
|  | Married | 207 (40.8) | 32 (53.3) | 49 (45.4) |
|  | Living as married | 36 (7.1) | 7 (11.7) | 3 (2.8) |
|  | Separated | 18 (3.6) | 2 (3.3) | 4 (3.7) |
|  | Divorced | 94 (18.5) | 3 (5.0) | 18 (16.7) |
|  | Widowed | 66 (13.0) | 2 (3.3) | 16 (14.8) |
| **Annual household income** |  |  |  |  |
|  | Less than $20,001 | 77 (15.2) | 9 (15.0) | 14 (13.0) |
|  | $20,001 to $35,000 | 120 (23.7) | 15 (25.0) | 20 (18.5) |
|  | 35,001 to $50,000 | 94 (18.5) | 11 (18.3) | 21 (19.4) |
|  | $50,001 to $75,000 | 99 (19.5) | 11 (18.3) | 28 (25.9) |
|  | $75,001 or more | 106 (20.9) | 13 (21.7) | 24 (22.2) |
|  | Prefer not to say | 11 (2.2) | 1 (1.7) | 1 (0.9) |
| **Clinic Distance** |  |  |  |  |
|  | Less than 5 miles | 204 (40.2) | 19 (31.7) | 36 (33.3) |
|  | Between 5 and 10 miles | 194 (38.3) | 32 (53.3) | 44 (40.7) |
|  | More than 10 miles | 109 (21.5) | 9 (15.0) | 28 (25.9) |
| **Area** |  |  |  |  |
|  | Urban | 130 (25.6) | 24 (40.0) | 24 (22.2) |
|  | Suburban | 245 (48.3) | 22 (36.7) | 55 (50.9) |
|  | Exurban | 15 (3.0) | 1 (1.7) | 4 (3.7) |
|  | Rural | 104 (20.5) | 9 (15.0) | 24 (22.2) |
|  | Blank answer | 13 (2.6) | 4 (6.7) | 1 (0.9) |

| Variable | Category | All Participants  N = 507 | RBPM Participation  n= 60 (11.8%) | RBPM Aware but No RBPM Participation  n= 108 (65.5%) |
| --- | --- | --- | --- | --- |
|  |  |  |  |  |
| **General Health Status** |  |  |  |  |
|  | Poor | 22 (4.3) | 1 (1.7) | 3 (2.8) |
|  | Fair | 120 (23.7) | 11 (18.3) | 24 (22.2) |
|  | Good | 238 (46.9) | 26 (43.3) | 46 (42.6) |
|  | Very good | 113 (22.3) | 18 (30.0) | 30 (27.8) |
|  | Excellent | 14 (2.8) | 4 (6.7) | 5 (4.6) |
| **Comorbidity** |  |  |  |  |
|  | Heart Condition | 0 (0.0) | 0 (0.0) | 0 (0.0) |
|  | Diabetes | 128 (25.2) | 18 (30.0) | 110 (24.6) |
|  | Depression or Anxiety | 203 (40.0) | 35 (58.3) | 168 (37.6) |
|  | Chronic kidney disease | 24 (4.7) | 1 (1.7) | 23 (5.1) |
|  | Other diseases | 99 (19.5) | 8 (13.3) | 91 (20.4) |
|  | No comorbidity | 137 (27.0) | 7 (11.7) | 130 (29.1) |
| **HTN History** |  |  |  |  |
|  | Less than 1 year | 22 (4.3) | 0 (0.0) | 3 (2.8) |
|  | 1 year – less than 2 years | 44 (8.7) | 16 (26.7) | 7 (6.5) |
|  | 2 years – less than 3 years | 63 (12.4) | 15 (25.0) | 9 (8.3) |
|  | 3 years – less than 4 years | 47 (9.3) | 10 (16.7) | 10 (9.3) |
|  | 4 years – less than 5 years | 44 (8.7) | 4 (6.7) | 14 (13.0) |
|  | 5 years or more | 287 (56.6) | 15 (25.0) | 65 (60.2) |
| **HTN Meds (mean, SD)** |  | 1.61 (0.96) | 1.65 (0.73) | 1.57 (0.76) |
| **Other Meds (mean, SD)** |  | 2.92 (2.83) | 2.42 (2.32) | 2.68 (3.33) |
| **BP under control** |  |  |  |  |
|  | Yes | 422 (83.2) | 53 (88.3) | 97 (89.8) |
|  | No | 46 (9.1) | 4 (6.7) | 5 (4.6) |
|  | Don’t know or Not sure | 39 (7.7) | 3 (5.0) | 6 (5.6) |
| **Systolic BP (mean, SD)** |  | 131.77 (18.15) | 129.24 (23.39) | 129.99 (16.47) |
|  |  |  |  |  |
|  |  |  |  |  |

| **Diastolic BP (mean, SD)** |  | 80.15 (11.80) | 81.64  (13.62) | 78.10  (9.80) |
| --- | --- | --- | --- | --- |
