# Supplementary material for: Behavioral Factors Related to Participation in Remote Blood Pressure Monitoring Among Adults With Hypertension: Cross-Sectional Study
Source: JMIR Form Res. 2024 Dec 23;8:e56954. doi: 10.2196/56954 (PMC11684531; doi:10.2196/56954)
Supplement: Multimedia Appendix 4 [file formative-v8-e56954-s004.docx]

Appendix 4. Remote BP monitoring (RBPM) strategies

| Variable | Category | Frequency (%), N = 60 |
| --- | --- | --- |
| RBPM Providers |  |  |
|  | Doctor | 47 (78.3) |
|  | Nurse | 22 (36.7) |
|  | Pharmacist | 4 (6.7) |
|  | Physician assistant | 12 (20.0) |
|  | Don’t know or unsure | 1 (1.7) |
| RBPM Frequency |  |  |
|  | Daily | 21 (35.0) |
|  | Several times a week | 24 (40.0) |
|  | Once a week | 9 (15.0) |
|  | One to three times a month | 2 (3.3) |
|  | Less than once a month | 4 (6.7) |
| RBPM Method |  |  |
|  | Text messages | 0 (0.0) |
|  | Email | 19 (31.7) |
|  | Electronic health record or patient portal | 21 (35.0) |
|  | Health Apps | 15 (25.0) |
|  | Phone call | 13 (21.7) |
|  | Automatic transfer from BP device to doctor | 10 (16.7) |
| RBPM Feedback type |  |  |
|  | None | 5 (8.3) |
|  | Readings received/acknowledgement | 30 (50.0) |
|  | Interpretation of readings as normal, high or low | 26 (43.3) |
|  | Changes in hypertension medication | 14 (23.3) |
|  | Changes in frequency of blood pressure monitoring | 11 (18.3) |
|  | Other feedback | 1 (1.7) |

RBPM: Remote blood pressure monitoring
