# Supplementary material for: Behavioral Factors Related to Participation in Remote Blood Pressure Monitoring Among Adults With Hypertension: Cross-Sectional Study
Source: JMIR Form Res. 2024 Dec 23;8:e56954. doi: 10.2196/56954 (PMC11684531; doi:10.2196/56954)
Supplement: Multimedia Appendix 5 [file formative-v8-e56954-s005.docx]

Appendix 5. Reasons for not participating and likelihood of participating in RBPM

| Variable |  | Category | (N = 447)  n (%) |
| --- | --- | --- | --- |
| Reasons for not participating in RBPM |  |  |  |
|  | 1 | My doctor has not asked me to do that | 247 (55.3) |
|  | 2 | I am not aware I can do that | 190 (42.5) |
|  | 3 | My blood pressure is under control | 92 (20.6) |
|  | 4 | I prefer face-to-face human interaction | 72 (16.1) |
|  | 5 | My doctor does not offer electronic communication means | 46 (10.3) |
|  | 6 | My doctor prefers to measure my blood pressure by himself/herself | 46 (10.3) |
|  | 7 | I do not have a blood pressure monitoring device | 46 (10.3) |
|  | 8 | I do not know how to do that/need training | 39 (8.7) |
|  | 9 | I do not measure my blood pressure | 38 (8.5) |
|  | 10 | I do not need to do that | 30 (6.7) |
|  | 11 | I do not have smartphone/tablet/computer | 5 (1.1) |
|  | 12 | Other reasons | 5 (1.1) |
|  | 13 | I do not have internet access | 2 (0.4) |
|  | 14 | I am too busy to do that | 1 (0.2) |
|  |  |  |  |
| Likelihood of participating in RBPM if offered |  |  |  |
|  |  | Very likely | 183 (40.9) |
|  |  | Somewhat likely | 152 (34.0) |
|  |  | Neither likely nor unlikely | 75 (16.8) |
|  |  | Somewhat unlikely | 18 (4.0) |
|  |  | Very unlikely | 19 (4.3) |

RBPM: Remote blood pressure monitoring
