# Supplementary material for: Behavioral Factors Related to Participation in Remote Blood Pressure Monitoring Among Adults With Hypertension: Cross-Sectional Study
Source: JMIR Form Res. 2024 Dec 23;8:e56954. doi: 10.2196/56954 (PMC11684531; doi:10.2196/56954)
Supplement: Multimedia Appendix 6 [file formative-v8-e56954-s006.docx]

Appendix 6. Technology ownership

| Variable | Category | All Participants  N = 507 | RBPM Participation  n= 60 (11.8%) | No RBPM Participation  n= 447 (88.2%) | P-value |
| --- | --- | --- | --- | --- | --- |
| Have Basic cellphone only |  |  |  |  | 0.004 |
|  | Yes | 89 (17.6) | 19 (31.7) | 70 (15.7) |  |
|  | No | 418 (82.4) | 41 (68.3) | 377 (84.) |  |
| Have Smartphone |  |  |  |  | 0.118 |
|  | Yes | 469 (92.5) | 59 (98.3) | 410 (91.7) |  |
|  | No | 38 (7.5) | 1 (1.7) | 37 (8.3) |  |
| Have Tablet Computer |  |  |  |  | ˂0.001 |
|  | Yes | 323 (63.7) | 51 (85.0) | 272 (60.9) |  |
|  | No | 184 (36.3) | 9 (15.0) | 175 (39.1) |  |
| Have Desktop or Laptop Computer |  |  |  |  | 0.324 |
|  | Yes | 440 (86.8) | 55 (91.7) | 385 (86.1) |  |
|  | No | 67 (13.2) | 5 (8.3) | 62 (13.9) |  |
| Have home BP monitoring device |  |  |  |  | ˂0.001 |
|  | Yes, I use it | 347 (68.4) | 58 (96.7) | 289 (64.7) |  |
|  | Yes, don’t use it | 52 (10.3) | 1 (1.7) | 51 (11.4) |  |
|  | No | 108 (21.3) | 1 (1.7) | 107 (23.9) |  |
| Home BP device payment |  |  |  |  | ˂0.001 |
|  | Paid by self | 286 (56.4) | 35 (58.3) | 251 (56.2) |  |
|  | Gifted | 25 (4.9) | 1 (1.7) | 24 (5.4) |  |
|  | Insurance paid | 78 (15.4) | 23 (38.3) | 55 (12.3) |  |
|  | Other | 10 (2.0) | 0 (0.0) | 10 (2.2) |  |
|  | No Home BP device | 108 (21.3) | 1 (1.7) | 107 (23.9) |  |
| Have Health Apps |  |  |  |  | ˂0.001 |
|  | Yes | 299 (59.0) | 56 (93.3) | 243 (54.4) |  |
|  | No | 208 (41.0) | 4 (6.7) | 204 (45.6) |  |

RBPM: Remote blood pressure monitoring, BP: Blood pressure
