# Supplementary material for: Behavioral Factors Related to Participation in Remote Blood Pressure Monitoring Among Adults With Hypertension: Cross-Sectional Study
Source: JMIR Form Res. 2024 Dec 23;8:e56954. doi: 10.2196/56954 (PMC11684531; doi:10.2196/56954)
Supplement: Multimedia Appendix 7 [file formative-v8-e56954-s007.docx]

Appendix 7. Technology use

| Variable | Category | All Participants  N = 507 | RBPM Participation  n= 60 (11.8%) | No RBPM Participation  n= 447 (88.2%) | P-value |
| --- | --- | --- | --- | --- | --- |
| Electronic communication with doctor or doctor’s office via email or internet |  |  |  |  | ˂0.001 |
|  | Yes | 377 (74.4) | 58 (96.7) | 319 (71.4) |  |
|  | No | 130 (25.6) | 2 (3.3) | 128 (28.6) |  |
| Electronic checking of medical tests |  |  |  |  | 0.041 |
|  | Yes | 381 (75.1) | 52 (86.7) | 329 (73.6) |  |
|  | No | 126 (24.9) | 8 (13.3) | 118 (26.4) |  |
| Achieving health goals with mHealth |  |  |  |  | ˂0.001 |
|  | Yes | 235 (46.4) | 51 (85.0) | 184 (41.2) |  |
|  | No | 272 (53.6) | 9 (15.0) | 263 (58.8) |  |
| Health decision making with mHealth |  |  |  |  | ˂0.001 |
|  | Yes | 277 (54.6) | 51 (85.0) | 226 (50.6) |  |
|  | No | 230 (45.4) | 9 (15.0) | 221 (49.4) |  |
| mHealth helps discussion with health care provider |  |  |  |  | ˂0.001 |
|  | Yes | 305 (60.2) | 56 (93.3) | 249 (55.7) |  |
|  | No | 202 (39.8) | 4 (6.7) | 198 (44.3) |  |
| Shared health information electronically with health care provider |  |  |  |  | ˂0.001 |
|  | Yes | 252 (49.7) | 56 (93.3) | 196 (43.8) |  |
|  | No | 255 (50.3) | 4 (6.7) | 251 (56.2) |  |
| Text messaging with doctor |  |  |  |  | ˂0.001 |
|  | Yes | 311 (61.3) | 54 (90.0) | 257 (57.5) |  |
|  | No | 196 (38.7) | 6 (10.0) | 190 (42.5) |  |

RBPM: Remote blood pressure monitoring
