# Supplementary material for: Behavioral Factors Related to Participation in Remote Blood Pressure Monitoring Among Adults With Hypertension: Cross-Sectional Study
Source: JMIR Form Res. 2024 Dec 23;8:e56954. doi: 10.2196/56954 (PMC11684531; doi:10.2196/56954)
Supplement: Multimedia Appendix 8 [file formative-v8-e56954-s008.docx]

| Appendix 8. Predictors of RBPM participation among those who are aware of RBPM using Firth's logistic regression | | | |
| --- | --- | --- | --- |
| Predictor Variables | Categories | Adjusted odds ratio (95%  Confidence interval) | P-values |
|  |  |  |  |
| **Age** |  | 1.00 (0.92, 1.07) | 0.94 |
| **Gender^a^** |  |  |  |
|  | Male | 0.90 (0.33, 2.43) | 0.84 |
| **Education level^b^** |  |  |  |
|  | Some college | 0.79 (0.01, 63.95) | 0.92 |
|  | College graduate or more | 23.72 (0.16, 3783.00) | 0.21 |
| **Race^c^** |  |  |  |
|  | American Indian or Alaska Native/ Asian/ Black or African American/ Other races | 0.59 (0.19, 1.76) | 0.35 |
| **Marital status^d^** |  |  |  |
|  | Married | 1.12 (0.33, 3.88) | 0.86 |
|  | Previously married | 0.36 (0.07, 1.67) | 0.19 |
| **Clinic distance from residence^e^** |  |  |  |
|  | Between 5 and 10 miles | 1.96 (0.69, 5.85) | 0.20 |
|  | More than 10 miles | 0.73 (0.18, 2.84) | 0.65 |
|  |  |  |  |
|  |  |  |  |
| **BP under control^f^** |  |  |  |
|  | Yes | 0.20 (0.02, 1.60) | 0.13 |
|  | Don’t know or unsure | 0.21 (0.01, 4.15) | 0.30 |
| **Have Tablet^g^** |  |  |  |
|  | Yes | 1.31 (0.40, 4.52) | 0.66 |
| **Have Smartphone^h^** |  |  |  |
|  | Yes | 0.80 (0.03, 30.10) | 0.89 |
| **Have Basic cellphone only^i^** |  |  |  |
|  | Yes | 2.74 (0.78, 10.81) | 0.12 |
| **Have Computer^j^** |  |  |  |
|  | Yes | 1.06 (0.26, 4.92) | 0.94 |
| **Have health Apps^k^** |  |  |  |
|  | Yes | 3.15 (0.60, 27.58) | 0.18 |
| **Electronic communication with doctor or doctor’s office via email or internet^l^** |  |  |  |
|  | Yes | 0.49 (0.05, 6.28) | 0.55 |
| **Sent or received SMS text message from doctor^m^** |  |  |  |
|  | Yes | 1.84 (0.49, 7.81) | 0.37 |
| **Shared health information from electronic device, tablet, or smartphone with health provider^n^** |  |  |  |
|  | Yes | 6.99 (1.62, 47.44) | 0.007 |
| **Made health decision with mHealth^o^** |  |  |  |
|  | Yes | 0.30 (0.05, 1.44) | 0.13 |
| **Achieved health goals with mHealth^p^** |  |  |  |
|  | Yes | 2.21 (0.56, 10.69) | 0.26 |
| **Have checked medical test results electronically^q^** |  |  |  |
|  | Yes | 0.49 (0.08, 2.48) | 0.40 |
| **Time since Hypertension diagnosis^r^** |  |  |  |
|  | Less than 1 year | 0.30 (0.002, 7.69) | 0.49 |
|  | 1 year - ˂ 2 years | 0.97 (0.17, 5.22) | 0.97 |
|  | 2 years - ˂ 3 years | 1.29 (0.29, 5.46) | 0.73 |
|  | 3 years - ˂ 4 years | 0.42 (0.07, 2.16) | 0.31 |
|  | 4 years - ˂ 5 years | 0.43 (0.07, 2.02) | 0.30 |
| **Age and Education interaction^s^** |  |  |  |
|  | Age and some college education | 0.98 (0.90, 1.06) | 0.60 |
|  | Age and college graduate or more | 0.93 (0.84, 1.01) | 0.08 |

Reference variables: a- female gender; b- less than college education; c- White race; d- never married; e- clinic distance less than 5 miles from residence; f- No response; g- No response; h- No response; i- No response; j- No response; k- No response; l- No response; m- No response; n- No response; o- No response; p- No response; q- No response; r- 5 years or more; s- Age and less than college education

RBPM: Remote blood pressure monitoring; BP: Blood pressure
