# Supplementary material for: Behavioral Factors Related to Participation in Remote Blood Pressure Monitoring Among Adults With Hypertension: Cross-Sectional Study
Source: JMIR Form Res. 2024 Dec 23;8:e56954. doi: 10.2196/56954 (PMC11684531; doi:10.2196/56954)
Supplement: Checklist 1 [file formative-v8-e56954-s009.docx]

Checklist for Reporting Results of Internet E-Surveys (CHERRIES)

| **Item Category** | **Checklist Item** | **Explanation** | **Page Number** |
| --- | --- | --- | --- |
| **Design** | Target population | Adults 18 years and above on Qualtrics® panel who have hypertension and are taking hypertension medications. Qualtrics® is a reputable experience management Web service provider that recruits verified people from various sources into their panel. | 4-5 |
|  | Sampling | Quota sampling based on age education. The proportions were based on a preliminary study on people with hypertension who participated in the 2018 Health Information National Trends Survey (HINTS) | 5 |
| **IRB (Institutional Review Board) approval and informed consent** | IRB approval | The University of Michigan IRB approved the study with the approval number HUM00205760. | 4 |
|  | Informed consent | An explanation of the survey with the statement of consent was presented in the beginning of the survey and only those who consented were allowed to continue the survey. | 6 |
|  | Data protection | The survey was domiciled at the University of Michigan Qualtrics website which is secure as well as Health Insurance Portability and Accountability Act (HIPPA) and Family Educational Rights and Privacy (FERPA) compliant. The authors of this study had no access to respondents’ emails and received an anonymized dataset containing no identifiable personal information |  |
| **Development and pre-testing** | Development and testing | The survey was developed by the authors based on what is obtainable in the literature regarding remote blood pressure monitoring, and adaptation of some questions from Health Information National Trends Survey (HINTS). The survey was first pilot tested among twelve volunteers from staff and graduate students and revised for clarity. The second pilot was done through the Qualtrics® panel to confirm content validity and reliability before the final launch. | 6-7 |
| **Recruitment process and description**  **of the sample having access**  **to the questionnaire** | Open survey versus closed survey | The survey was a closed survey only accessible to those who got the survey link through their emails. |  |
|  | Contact mode | The survey link was sent by email to Qualtrics® panel members. Only those with the link were able to access the survey. |  |
|  | Advertising the survey | The survey was advertised to Qualtrics® panel members through email invitation. |  |
| **Survey administration** | Web/E-mail | The survey was housed in the University of Michigan Qualtrics® website. Only those with the link were able to access the survey, and their responses were recorded automatically on the website. |  |
|  | Context | The University of Michigan (U-M) Qualtrics® is within the University of Michigan Information and Technology Services. Qualtrics at U-M is an online survey platform available to U-M faculty, staff, and students to use for university-related work. It is HIPAA and FERPA compliant. Only those with approved access link or login ID can access the survey. |  |
|  | Mandatory/voluntary | Participation in the survey was voluntary |  |
|  | Incentives | Qualtrics® offers individualized incentive to their panel members to participate in surveys. |  |
|  | Time/Date | The survey was in the field from November to December 2021. | 6 |
|  | Randomization of items or questionnaires | The questions were organized in sections for logical flow and structure. There was no need for randomization because the respondents could only access the questionnaire once. |  |
|  | Adaptive questioning | Conditioning and routing were used where relevant. |  |
|  | Number of items | One questionnaire item was displayed per page |  |
|  | Number of screens (pages) | The questionnaire was distributed over 64 pages. A progress bar ranging from 0 to 100% was provided at the top of the pages to help respondents see their progress with the survey completion. |  |
|  | Completeness check | Completeness check was done during the analysis. Responses were required for the consent, screening and routing questions. |  |
|  | Review step | A back button was provided for respondents to review answers if needed. |  |
| **Response rates** | Unique site visitor | The survey system used IP addresses to determine unique visitor |  |
|  | View rate | Not applicable |  |
|  | Participation rate | Not applicable |  |
|  | Completion rate | Completion rate was 100% because the Qualtrics® system only keeps records of those who complete the survey. |  |
| **Preventing multiple entries from the same individual** | Cookies used | Cookies were not used |  |
|  | IP Check | IP address was not required from respondents, but the survey system collected IP addresses and that was helpful in confirming that there were no duplicate entries from same address. |  |
|  | Log file analysis | The survey data was manually scanned to detect and delete meaningless entries during and at the end of the data collection. |  |
|  | Registration | The survey system was set up such that once a respondent completes a survey, it cannot be displayed a second time. |  |
| **Analysis** | Handling of incomplete questionnaires | The survey system was set up such that only completed questionnaires were recorded. |  |
|  | Questionnaires submitted with an atypical time stamp | There was no cut-off point for filling the questionnaire. |  |
|  | Statistical correction | No statistical correction procedures or weightings were used. |  |
